# Correspondence

**Published:** 1854-07

**Authors:** W. W. Granger


### Correspondence.

FAIRMOUNT, Marion county, Va., April 29, 1854.

DR. JAMES TAYLOR: *Dear Sir*:— * * * * * I have read the "Register" with much pleasure, and should not be willing to part with it for a trifle—valueing it none the less for a brief acquaintance which you may or may not remember to have occurred at Monongahela City, while yourself and family were on a visit there in 1852.

You will see, from the enclosed card, that though I have located, and returned to the practice of my regular profession—Dentistry—I have not altogether given up the occupation in which I was then engaged. What is worse yet—*don't* tell Prof. Smith, for I see his Valedictory is down on professional double-barrels, or "jacks of all trades," as he calls them,—I have, besides, the half editorship of a country weekly to attend to. You can readily imagine I am at no loss for employment from half past six, A. M., till twelve at night. But to be honest, a single occupation would be preferable, by all means, if one alone would furnish employment and a livelihood. While I have a high regard for Prof. Smith's acknowledged ability, and am decidedly pleased with most points of his Valedictory, there are two points on which he must pardon me for a difference of opinion. The first of these is the implied idea that a Dentist should, under no circumstances, combine other employments with those of his profession. If he will imagine a Dentist, tied by matters beyond his control, at a point where a sufficient present practice for a livelihood is unattainable, but the prospect good for an eventually lucrative field, would he have him, for professional dignity's sake, starve out, rather than use his vacant time in some other honorable calling? Let him suppose, for instance, a Dentist located, and *bound to stay*, for some years at least, in a county of some 15,000 inhabitants, with capacity for maintaining three times that number—its resources just beginning to develop, and the people in the transition stage from the one-room-log-house degree to the many-roomed, three-story-brick-and-frame-house stage of civilization. Let him suppose that Dentist to have an honorable standard of professional excellence, by which he aims to measure every operation he attempts, and to have a scale of fees in accordance. Then suppose him to have been *liberally* preceded by a class of operators who would hunt work from house to house, as a huckster would chickens, and when found, would "pull" teeth at 12½ cents each, or plug them (with sheet lead or paste) at 25 to 50 cents, and the public confidence in Dentistry such as under these circumstances might be expected. What would Prof. Smith have a body do in such a case? I should not think it dishonorable or non-professional under suoh circumstances, after opening an office for such practice as might offer, and doing it to the best of my ability when it did come, to use the balance of my time in any other honorable and remunerative pursuit, waiting patiently for the increase of practice, which I have always found sure to follow operations well performed at fair prices, and dropping other pursuits, or handing them over to proxies. as Dental practice increased. Other things can be done by proxy: he who would acquire reputation as a Dentist, must perform every operation for which he is responsible.

Secondly, I think the Professor was a little too fast in assuming that a man cannot excel in more than one pursuit. His assertion that "you will invariably find that those who are doing more or less at several trades or professions, are far from being good at either," [see p. 172, *Dental Register*, April, 1854] is not, I think, entirely supported by the biography of past generations, or that of the present. At all events, there are those who hold the opinion that Michael Angelo was not "far from good" as a painter, not bad as a sculptor, nor without claims to distinction as an architect. At the same time, his reputation in history and polite literature, left an impression on those branches which is not yet rubbed out; and he was far from unknown in both the state and religious affairs of his time. There is room to doubt whether even great Cæsar was greatest as author, general, orator or statesman. Had not Milton's greatness in the political world been eclipsed by his *greater* greatness in the sphere of song, his political career alone would have made him still remembered as one of that generation's most influential men. Take from the brow of Ireland's Sheridan any four of its quintuple crowns, and he would still stand amongst men an intellectual king —like Israel's Saul, "head and shoulders above his fellows." Actor, author, lawyer, parliamentarian and orator, as he was at once, I do not suppose his eminence in either department will be questioned. If it is, it will be by men of less attainments, or ability either, than are possessed by Professor Smith. He certainly will not consider England's Bulwer the less a diplomatist because of confessed celebrity as a dramatist and novel-writer. Lord Bacon took "*all* knowledge for his profession," and was not "far from good" in *many* departments, or the mass of learned men, then and since, have been mistaken in their estimates of him. Marryatt's success as a novelist did not hinder his skill as a navigator. Clay, Calhoun and Webster, were not considered "far from good" as lawyers, and were not altogether unknown as statesmen. If Prof. Smith's attention has not been *very* exclusively given to Dentistry and its "collateral studies," he may have heard of them in both capacities, and perhaps will not be surprised by hearing that each was eminent in a third pursuit, not generally ranked as "collateral" to either of the others—I mean agriculture. Washington has been heretofore assigned some excellence in four somewhat anti-collateral callings—farming, fighting, legislation and the Chief Magistracy—besides which, he did some surveying in earlier life, and was not so "far from good" at that, but that the lines he run almost a century ago have been standards ever since. It would be no hard task to mention, of the present generation, several who in circus parlance could be termed "two and four horse riders." To say nothing of Barnum, whose eminent success is not a breath more notorious than the seven-handedness with which he has struggled to achieve it, we will take one Western man—Pike, of Arkansas—who is known everywhere as an able lawyer, poet, artist, legislator, novel-writer (I believe) and political stump-speaker. Not to slight our own profession, or its members, Dr. Hullihen, of Wheeling, may be cited as one who is not *so* "far from good," but that he is eminently successful in a much wider field than I understand the Professor's language to assign any one man the privilege of working. Optical, Obstetrical and General Surgeon, Surgeon and Mechanical Dentist; managing, in conjunction with his partner, an office devoted to the latter speciality, and an infirmary for the other classes of operations, besides a laboratory, where he makes his own teeth, plate, foil and instruments.

In the name of all these easily multiplied exceptions to his theory, I protest against any standard of professional brotherhood, based on his assumption that "you will *invariably* find that those who are doing more or less at several trades or professions, are far from being good at either." Those who, by the necessity of surrounding circumstances, are forced to pursue jointly two or more callings for a time, may stand on the right of that necessity to do so, while it exists. Those whose genius and abilities fit them for all or any of a dozen callings, have, in that fact, a diploma, written by the hand of Omnipotence, warranting their pursuit, when circumstances call, of either or all combined. Still, it is advisable for most men, under most circumstances, to pursue a single calling. The frequent vexations and incessant toil of several combined, are enough to keep most men from attempting more than one; and if excellency in all is aimed at, still harder labor, still shorter sleep, and yet more troubled dreams, greater risk to health, and the certain earlier coming of old age and death, are the prices, brother Dentist, it will cost. While I protest against Professor Smith's doctrine as a *law* —a test of professional brotherhood, I dare not do else than recommend it as *advice*, which ninety-nine in the hundred had best follow.

W. W. GRANGER.

[REMARKS.—The above communication from Dr. Granger we insert with pleasure, and are pleased to hear that he has got back to his legitimate profession—Dentistry. We would, however, give him due credit as a Daguerrotypist; yet with his success in this, and all the splendid cases of exceptions to which he has alluded—which we regard as exceptions to a general rule—we believe if he were placed on the same stand-point with Prof. Smith, that he would like his doctrine quite as much as his admirable address.

We hope that Dr. Granger will e'er long so introduce Dentistry into Fairmount, as to be obliged to employ proxies for the other duties which press upon him.]—*Ed. Dental Register.*

